# Kynurenines in the Pathogenesis of Peripheral Neuropathy During Leprosy and COVID-19

**DOI:** 10.3389/fcimb.2022.815738

**Published:** 2022-02-24

**Authors:** Jéssica Araujo da Paixão de Oliveira, Mariana Martins de Athaide, Atta Ur Rahman, Mayara Garcia de Mattos Barbosa, Marcia Maria Jardim, Milton Ozório Moraes, Roberta Olmo Pinheiro

**Affiliations:** ^1^Leprosy Laboratory, Oswaldo Cruz Institute, Oswaldo Cruz Foundation, Rio de Janeiro, Brazil; ^2^Department of Surgery, University of Michigan, Ann Arbor, MI, United States; ^3^Department of Neurology, State University of Rio de Janeiro, Rio de Janeiro, Brazil

**Keywords:** kynurenine pathway, peripheral neuropathy, leprosy, COVID-19, tryptophan

## Abstract

Inflammatory disorders are associated with the activation of tryptophan (TRYP) catabolism *via* the kynurenine pathway (KP). Several reports have demonstrated the role of KP in the immunopathophysiology of both leprosy and coronavirus disease 19 (COVID-19). The nervous system can be affected in infections caused by both *Mycobacterium leprae* and SARS-CoV-2, but the mechanisms involved in the peripheral neural damage induced by these infectious agents are not fully understood. In recent years KP has received greater attention due the importance of kynurenine metabolites in infectious diseases, immune dysfunction and nervous system disorders. In this review, we discuss how modulation of the KP may aid in controlling the damage to peripheral nerves and the effects of KP activation on neural damage during leprosy or COVID-19 individually and we speculate its role during co-infection.

## Introduction

During severe COVID-19, there is an increase in the inflammatory status caused by a cytokine storm (Hu et al., 2021; Yang et al., 2021). In leprosy, acute inflammatory episodes, named reactional episodes, are characterized by a sudden increase in pro-inflammatory mediators and an intensification of neural damage (Serrano-Coll et al., 2018). The increase in pro-inflammatory mediators in COVID-19 and in leprosy may increase the activity of the enzyme Indoleamine 2,3-dioxygenase 1 (IDO1), which results in the production of kynurenine metabolites (Belladonna and Orabona, 2020; Turski et al., 2020). The catabolism of tryptophan (TRYP) to the immunosuppressive and neuroactive kynurenines is a key metabolic pathway, known to regulate immune responses and neurotoxicity (Harden et al., 2016; Sundaram et al., 2020; Siska et al., 2021).

Leprosy is an infectious disease caused by the intracellular bacteria *Mycobacterium leprae* or by *M. lepromatosis*. These bacteria exhibit an affinity for Schwann cells, leading to peripheral nerve damage (Hess and Rambukkana, 2019; Rambukkana, 2000; Rambukkana, 2001). Although the new coronavirus, SARS-CoV-2, and the disease it causes, COVID-19, is associated with respiratory system damage, it can also be associated with disorder of the central (CNS) and peripheral nervous system (PNS) (Andalib et al., 2021; McFarland et al., 2021).

Some PNS manifestations observed in patients with COVID-19 are as follows: Guillain-Barré syndrome, cranial polyneuritis, neuromuscular junction disorders, neuro-ophthalmological disorders, neurosensory hearing loss, and dysautonomia (Andalib et al., 2021). Neuropilin-1 and -2 (NRP1 and NRP2) are entry factors for SARS-CoV-2, which could induce changes in nociceptor (‘pain receptor’) excitability (McFarland et al., 2021). Pain is also a symptom that may occur in leprosy patients. Pain may be nociceptive, neuropathic or mixed (Angst et al., 2020). There is evidence that the cytokine profile in COVID-19 contributes to the promotion of pain and also improves pain states (McFarland et al., 2021; Weng et al., 2021), and that SARS-CoV-2 infection may worsen neural damage or neural pain in leprosy patients.

## Host–Cell Interaction in the Context of Leprosy Neuropathy

Leprosy neuropathy is considered the most common chronic peripheral degenerative disease of infectious origin, as *M. leprae* is one of the few bacterial pathogens with the ability to enter the hemato-nerve, invade the PNS, and establish infection (Scollard et al., 2015; Serrano-Coll et al., 2018). *M. leprae* accesses the PNS by preferentially invading glial cells, such as Schwann cells (SCs) (Scollard, 2008; Brown et al., 2012) and induces nerve damage by direct and indirect mechanisms (Serrano-Coll et al., 2018) (**Table 1**).

**Table 1:** Cellular and molecular mechanisms of *M. leprae* interaction with peripheral nerve cells.

Reference	Study Type	Objectives	Experimental Model	Results	Conclusion
**1**. (Díaz Acosta et al., 2018)	*In vitro*	PGL-1 help in *M. leprae* entry and survival in Schwan cell.	ST8814 Human Schwann cells line	PGL-1 induced the expression of MR/CD206 and PPARγ in infected cell that may help in *M. leprae* recognition, entry and survival. CD206/PPARγ crosstalk also induce lipid droplet production and accumulation in Schwan cell.	PGL-1 of live *M. leprae* induces more adherence and internalization then dead *M. leprae*.
**2**.(Nogueira et al., 2018)	*In vitro* and *In vivo*	Effect of *M. leprae* on neurotrophins expression and their role in leprosy neuropathy.	Human Schwann cells and Mice model	Downregulation of neurotrophins such as NT-4, NGF and BDNF mRNA in *M. leprae* treated hSC while upregulation of NT-3 in *M. leprae* treated mice. Imbalance of these factor may have role in nerve impairment.	*M. leprae* may involve in neurotrophins regulation that may induce nerve degeneration or repair.
**3**. (Hagge et al., 2002)	*In vitro*	Maintaining viability of *M. leprae* in Schwan cells, Schwann cells and Schwann cell–axon interactions in co-cultures. Effect of temperature on the viability of *M. leprae*.	Rat Schwann cells	Schwan cell maintain 56% viability at 33˚C for 3 weeks and altered morphology and genes expression that encoding cellular adhesion molecules but were capable of cellular interaction. Schwann cell neuron cocultures, infected after myelination and no morphological changes were found in myelin architecture at 33 ˚C after 30 days of incubation with 53% viability of *M. leprae*.	This model will be helpful to study the effects of *M. leprae* on Schwann cells and Schwann cell-neuron interactions and maintain the culture for long time at 33˚C.
**4**. (Oliveira et al., 2003)	*In vitro*	Human Schwann cells are susceptible to cell death by activation of Toll-like receptor 2 (TLR2).	ST8814 Human Schwann cells line	TLR2 were expressed at moderate levels in comparison to MHC-I but at higher levels to MHC-II. The 19-kDa lipoprotein of *M. leprae* trigger apoptosis and promote inflammation. The frequency of apoptotic cells increases in presence of TLR2 and 19-kDa lipoprotein of *M. leprae*.	TLR2 ligation induce apoptosis of human Schwann cell and cause nerve damage by host immune response.
**5**. (Madigan et al., 2017)	*In vivo*	Macrophage responses to *M. leprae* specific PGL-1 trigger demyelination and nerve damage in leprosy.	Zebra fish larva model	Infected macrophages patrolling toward and come in close contact with axon, cause demyelination. PGL-1 confer the macrophage neurotoxic response by inducing RNS and ROS production, causing axonal and mitochondrial damage that leads to demyelination.	*M. leprae* PGL-1 induce macrophage response that are greatly involve in demyelination and nerve damage.
**6**. (Mietto et al., 2020)	*In vitro*	Myelin breakdown favor *M. leprae* survival.	Mice Schwann cells	*M. leprae* infect Schwan cell and accelerate myeline destruction *via* mechanism called myelinophagy that leads to increase lipid droplet formation which are major contributor for *M. leprae* persistence in Schwann cells.	Myelin breakdown induces lipid droplet production, providing protective lipid-enriched shelters for *M. leprae* inside Schwan cell.
**7**. (Masaki et al., 2013)	*In vitro* and *In vivo*	Understood how initial colonization of *M. leprae* in Schwann cells subsequently could lead to the spread of infection to other tissues.	Mouse primary Schwann cells and Mice model	The leprosy bacterium hijacks this property to reprogram adult Schwann cells, its preferred host niche, to a stage of progenitor/stem-like cells (pSLC) of mesenchymal trait by downregulating Schwann cell lineage/differentiation-associated genes and up- regulating genes mostly of mesoderm development. Reprogramming accompanies epigenetic changes and renders infected cells highly plastic, migratory, and immunomodulatory.	pSLC promotes bacterial spread by two mechanisms: direct differentiation to mesenchymal tissues, including skeletal and smooth muscles, and formation of gran- uloma-like structures and subsequent release of bacteria-laden macrophages.
**8**. (Masaki et al., 2014)	*In vitro*	The role of innate immune response in cellular reprogramming and the initiation of neuropathogenesis during *M. leprae* infection.	Mouse primary Schwann cells	*M, leprae* hijacks induce a large number of immune-related genes comprising mostly innate immunity and chemokine-associated genes right from the very early stage of Schwann cell infection and peaking in their expression when Schwann cells have changed their cell identity to pSLCs.	*M. leprae* induces the expression of a variety of genes related to innate immunity in Schwann cells strains in the early stage of infection, even before there were gene modifications associated with reprogramming in pSLC.
**9**. (Petito et al., 2013)	*In vitro* and *Ex vivo*	Effects of *M. leprae* on Schwann cells TGF-β1 transcription and secretion, the effects of exogenous TGF-β1on α-SMA expression, morphology, and matrix-producing myofibroblasts deposition *in vitro* and correlated the findings with those in nerve biopsies of leprosy patients.	ST8814 Human Schwann cells line	Schwann cells transdifferentiates into extracellular matrix-producing myofibroblasts under the stimulus of TGFβ-1, by a mechanism in which *M. leprae* is the inducer of this event.	*M. leprae* infected Schwann cells undergo phenotypical changes and even death as a result of inflammatory mediators, leading them to secrete ECM that contributes to progressive nerve fiber loss and fibrosis.
**10**. (Oliveira et al., 2005)	*In vitro*	Determine if cytokines and *M. leprae* were capable of triggering human Schwann cell apoptosis and, as such, of contributing to nerve damage in leprosy.	ST8814 Human Schwann cells line	Presence of TNF-Rs and TGF- RII on the Schwann cells membrane and the shedding of TNF-Rs during the culture period. TNFα/TGF-β1 combination as well as *M. leprae* infection triggered an increase in the apoptosis rate in the cultured Schwann cells. Moreover, qRT-PCR revealed that *M. leprae* upregulated the expression of such cytokines and their receptors on the Schwann cells line.	Induction of Schwann cells death, can pro- vide an effective mechanism of ongoing tissue injury during *M. leprae* infection, which, in turn, may be further modulated by cell–cell interaction and cytokine production both *in vitro* and *in vivo*.
**11**. (Medeiros et al., 2016)	*In vitro* and *Ex vivo*	Effect of *M. leprae* on Schwann Cell Glucose Metabolism.	ST8814 Human Schwann cells line	*M. leprae* infection was able to modulate Schwann cell glucose metabolism, generating a marked increase in glucose uptake and the PPP oxidative cycle key enzyme G6PDH. In addition, *M. leprae* infection also reduced mitochondrion membrane potential and lactate release by Schwann cells. These alterations resulted in free-radical control.	*M. leprae* could modulate host cell glucose metabolism to increase the cellular reducing power generation, facilitating glutathione regeneration and, consequently, free-radical control.
**12**. (Borah et al., 2019)	*In vitro*	Measure carbon metabolism of *M. leprae* in its primary host cell, the Schwann cell.	ST8814 Human Schwann cells line	*M. leprae* utilizes host glucose pools as the carbon source to biosynthesize the majority of its amino acids. The anaplerotic enzyme phoenolpyruvate carboxylase is required for this intracellular diet of *M. leprae*.	Intracellular *Mycobacterium leprae* utilizes Host Glucose as a Carbon Source in Schwann Cells to biosynthesize the majority of its amino acids.
**13**. (Andrade et al., 2016)	*Ex vivo*	Envelopment of Inflammatory Cytokines in Focal Demyelination in Leprosy Neuritis	Human model	*ML* is capable of contributing to a TNF-mediated response by inducing mTNF expression and upregulating TNFR1, thus rendering Schwann Cells more sensitive to the exogenous TNF levels in the nerve, which likely originates from resident macrophages in the early stages of injury and, later, from inflammatory cells. Moreover, *M. leprae* induces IL-23 secretion in Schwann Cells	*M. leprae* may contribute to TNF-mediated inflammation and focal demyelination by rendering SCs more sensitive to TNF within the nerves of patients with leprous neuropathy.
**14**. (Rambukkana et al., 2002)	*In vitro* and *In vivo*	Measure the functional consequences of early *M. leprae* interaction with peripheral nerves.	Schwann cell - DRG neuron coculture	*M. leprae* induced rapid demyelination by a contact-dependent mechanism in the absence of immune cells in *an in vitro* nerve tissue culture model and in Rag1-knockout (Rag12/2) mice. Myelinated Schwann cells were resistant to *M. leprae* invasion but undergo demyelination upon bacterial attachment, whereas nonmyelinated Schwann cells harbor intracellular *M. leprae* in large numbers.	*M. leprae* propagates a nonmyelinating phenotype by inducing demyelination and nerve injury in myelinated Schwann cells in the early phase of infection, a novel bacterial survival strategy in the nervous system.
			Mice model		
**15**. (Tapinos et al., 2006)	*In vitro*	Identify the molecular events that occur in the early phase before the progression of the leprosy.	Primary Schwann cells and myelinated Schwann cell–neuron cocultures.	*M. leprae*, by directly binding to and activating ErbB2 without ErbB3 heterodimerization, and thus bypassing neuregulin-ErbB3–mediated ErbB2 phosphorylation, induces excessive downstream Erk1/2 signaling and subsequently causes demyelination.	Therapeutic interventions targeted to block kinase activity of ErbB2 may have the potential to prevent nerve degeneration in leprosy and other demyelinating diseases at an early stage before the progression of these neurodegenerative diseases.
**16**. (Oliveira et al., 2010)	*In vitro*	Effects of TNF and *M, leprae* in leprous neuropathy.	ST8814 Human Schwann cells line and *Ex vivo*	*M. leprae* and TNF induced upregulation of MMP-2 and MMP-9 and increased secretion of these enzymes in cultured ST88-14 cells. The effects of TNF and *M. leprae* were synergistic. Nerves with inflammatory infiltrates and fibrosis displayed higher TNF, MMP-2, and MMP-9 mRNA than controls. Leprous nerve biopsies with no inflammatory alterations also exhibited higher MMP-2 and MMP-9. The biopsies with endoneurial inflammatory infiltrates and epithelioid granulomas had the highest levels ofMMP-2 and MMP-9 mRNA detected.	*M. leprae* and TNF may directly induce Schwann cells to upregulate and secrete MMPs regardless of the extent of inflammation in leprous neuropathy.

Previous studies have shown that cell reprogramming occurs during the interaction between *M. leprae* and SCs. Masaki et al. (2013) found that *M. leprae* adult SCs undergo reprogramming to be converted into cells that are similar to those of the neural crest, known as progenitor/stem-like cells (pSLCs), contributing to the systemic spread of the bacillus. Moreover, Petito et al. (2013) showed that SCs transdifferentiate into the extracellular matrix (ECM)-producing myofibroblasts under the stimulus of transforming growth factor beta-1 (TGF-β1). *M. leprae* has been reported to induce this event, since the bacillus has the ability to increase the expression of TGF-β1 and its receptors in SCs (Oliveira et al., 2005), which can lead to the progression of neural damage by promoting the fibrosis process. Despite these findings, the details of the signaling pathways involved in host cell reprogramming by the pathogen have yet to be elucidated.

The literature has provided us with some evidence indicating a strong relationship between host cell metabolism and the inflammatory response against *M. leprae*, and how they can influence each other (de Macedo et al., 2020; Oliveira J. A. P. et al., 2021). *In vitro* studies demonstrated that *M. leprae* is able to induce lipid body biogenesis in macrophages and SCs, favoring bacilli survival inside these host cells, by a mechanism that is dependent on the innate immune receptors, Toll-like receptor 6 (TLR6) and TLR2 (Mattos et al., 2010; Mattos et al., 2011). Furthermore, the abundant *M. leprae* surface molecule, phenolic glycolipid 1 (PGL-1), can induce the expression of the mannose receptor (MR/CD206) in infected SCs by a mechanism that is dependent on proliferator-activated receptor gamma (PPARγ). Lipid droplets are associated with mycobacterial survival and are pivotal in bacterial pathogenesis (Díaz Acosta et al., 2018). In addition, Medeiros et al. (2016) observed that *M. leprae* is able to subvert SC metabolism *in vitro* by modulating glucose uptake, increasing the generation of reducing power and controlling the production of free radicals, which led to increased intracellular viability in the bacillus. Furthermore, *M. leprae* amino acids are derived from host glucose pools, which provide the carbon source (Borah et al., 2019).

*M. leprae* infection induces demyelination after contact with myelinated fibers, leading to the breakdown of the myelin sheath, the fatty tissue that protects the nerves (Rambukkana et al., 2002; Tapinos et al., 2006). Mietto et al. (2020) demonstrated that *M. leprae* infection in SCs of mice is capable of inducing myelin sheath breakage *via* myelinophagy, with the formation of myelin ovoids. These data reinforce the importance of the lipid metabolism for the persistence and maintenance of the bacillus in the peripheral nerve, consequently favoring the destruction of nerve fibers.

In the neuroinflammatory context, SCs have important immune functions in response to infection and in the production of mediators secreted by inflammatory cells present in the lesion. Pattern recognition receptors (PRRs), such as TLR2, are known to be expressed in the SCs of skin lesions of leprosy patients. Furthermore, *M. leprae* and its ligands induce SC death *via* apoptosis, by a mechanism dependent on TLR2 and tumor necrosis factor (TNF) (Oliveira et al., 2003; Oliveira et al., 2005). TNF and *M. leprae*, synergistically, can change the SC phenotype *in vitro*, leading to the production of proteases such as metalloproteinase 2 and 9 (MMP2 and MMP9). It was observed that nerves from patients with leprosy neuropathy have a higher expression of these proteases, in addition to TNF (Oliveira et al., 2010). These proteases contribute to myelin degradation and increase the extent of neural damage, contributing to the fibrosis process. TNF involvement was also seen in reactive patients with neuritis, who showed an increased expression of TNF, the TNF receptor, and TNF-converting enzyme in nerve biopsies (Andrade et al., 2016).

Innate immune response components, such as cytokines and chemokines, are widely associated with peripheral nerve damage in leprosy (Oliveira et al., 2003; Medeiros et al., 2015; Andrade et al., 2016). Masaki et al. (2014) showed that *M. leprae* induces the expression of a variety of genes related to innate immunity in SCs in the early stage of infection, even before the gene modifications associated with reprogramming in pSLC. According to Madigan et al. (2017), infected macrophages produce nitric oxide synthase (iNOS) leading to demyelination and axon damage by a mechanism mediated by mycobacterial surface molecule PGL-1.

## Co-Infection Leprosy and COVID-19

COVID-19 brought additional challenges to the health system of several countries and may have had effects on other prevalent diseases around the world, such as leprosy (Rathod et al., 2020; Matos et al., 2021). Despite many leprosy institutions remaining open and offering services during the pandemic, providing leprosy diagnosis, multidrug therapy (MDT), and leprosy reaction medications, many patients were unable to travel to these centers (de Barros et al., 2021). Mahato et al. (2020) emphasized the impact of the current scenario on leprosy-affected individuals in Nepal. Due the natural progress of leprosy, affected individuals require long-term follow-up. The measures that were recommended to reduce the transmission of SARS-CoV-2 likely created barriers to health services for these leprosy patients, disrupting the disease management.

A recent finding suggests that co-infection of COVID-19 and leprosy, especially the multibacillary form of the disease, can lead to serious conditions or even death in these patients, mostly in men, the elderly, and those with non-communicable diseases (Santos et al., 2021). However, epidemiological studies are needed to determine the real impact of COVID-19 on leprosy patients.

Although the immunological responses to SARS-CoV-2 infection have been extensively studied, there is no consensus regarding the mechanisms associated with its variations and levels of severity, as well as its interaction with other diseases. As in leprosy, COVID-19 is known to present with a variable response according to the individual. The intense release of inflammatory mediators resulting from the cytokine storm can have negative impacts on leprosy patients; thus, it has been suggested that cytokine responses in SARS-CoV-2 infection may alter the clinical outcome of leprosy (Antunes et al., 2020). Recently, Cerqueira et al. (2021) demonstrated that leprosy patients are more vulnerable to COVID-19 because they have more frequent contact with SARS-CoV-2-infected patients, perhaps due to the social and economic aspects.

IL-6 is elevated in patients with COVID-19 (Santos Morais Junior et al., 2021) and is positively correlated with the severity of COVID-19 symptoms (Kirtipal and Bharadwaj, 2021). IL-6 is an important pro-inflammatory mediator, involved in the activation of immune cells in the brain, which contributes to injury of the brain tissue (Espíndola et al., 2021), and is a marker of neuropathic pain in leprosy (Angst et al., 2020). IL-6 has been described as an important immunological stimulus triggering leprosy reactions, and thus increase the risk of developing leprosy neuropathy (Sales-Marques et al., 2017; Tió-Coma et al., 2019).

According to Morais et al. (2021), patients with *M. leprae*/SARS-CoV-2 co-infection showed increased IL-6 gene expression; moreover, the median disability grade was higher for *M. leprae*/SARS-CoV-2-co-infected patients than for patients with leprosy alone, even more than 30 days after the onset of COVID-19. The World Health Organization (WHO) classifies leprosy disability based on the WHO grading system, such as grade 0: normal sensation, no visible impairments, grade 1: impaired sensation, no visible impairments due to leprosy, and grade 2: visible impairments/deformity. Thus, this result suggests that SARS-CoV-2 co-infection may influence the development of neuropathy in leprosy by a mechanism involving increased IL-6 expression. The IL-6 polymorphism was proposed for use as an indicator of severity in COVID-19 patients in the Korean population (Kirtipal and Bharadwaj, 2021). In addition, clinical studies have shown that single-nucleotide polymorphisms (SNPs) in the IL-6 gene are associated with leprosy reactions (Sales-Marques et al., 2017). Thus, *M. leprae*/SARS-CoV-2 co-infection may trigger a higher-grade pro-inflammatory state, and the use of IL-6 inhibition to prevent neural damage might be a promising treatment strategy.

SARS-CoV-2 infection in leprosy patients has raised important questions about the incidence and/or severity of the reactional episodes (Antunes et al., 2020; Santos et al., 2021). The two main types of leprosy reaction are referred to as a type-1 reaction or reversal reaction (RR) and type-2 reaction or erythema nodosum leprosum (ENL), each with its own distinct characteristics. Cytokine storms and high levels of systemic inflammatory mediators have been described in ENL patients. Whether the *M. leprae*/SARS-CoV-2 co-infection could trigger the onset of ENL by enhancing the neurological damage, leading to physical disabilities, remains to be seen (Schmitz and Dos Santos, 2021).

Despite concerns about the severity of *M. leprae*/SARS-CoV-2 co-infections, curiously, the drugs used in MDT have been associated with a favorable outcome for COVID-19 patients (Arora et al., 2021; de Barros et al., 2021; Saxena et al., 2021). Clofazimine, an anti-leprosy drug, may have a role in the control of SARS-CoV-2 and MERS-CoV in the Middle East since it has been demonstrated to antagonize SARS-CoV-2 replication in multiple *in vitro* and ex vivo human systems, as well as in a hamster model of SARS-CoV-2 pathogenesis (Yuan et al., 2021). However, Cerqueira et al. (2021) described that the use of corticosteroids, thalidomide, pentoxifylline, clofazimine, or dapsone or BCG vaccination did not affect the occurrence or severity of COVID-19.

## SARS-CoV-2: Host–Cell Interaction and Immune Modulation

COVID-19 has affected more than 200 million people worldwide since the first case was detected. Although the pulmonary complications are profound, neurological manifestations were also observed (Bridwell et al., 2020; Andalib et al., 2021).

Previous findings have demonstrated that SARS-CoV-1, MERS-CoV, and OC43 coronaviruses present neurotropism (Iadecola et al., 2020). SARS-CoV-2 can reach the brain after infecting nasal cells. A previous study demonstrated that it could cause inflammation and demyelination in cells from CNS (Zoghi et al., 2020).

Headache, epilepsy, and disturbances of consciousness are observed in some patients with COVID-19, and loss of smell or taste is a frequent related symptom (Guastalegname and Vallone, 2020; Hopkins et al., 2020).

There is evidence that the SARS-CoV-2 glycoproteins bind to angiotensin-converting enzyme 2 (ACE-2) receptors to enter the host cell. The binding of the viral spike (S) protein to ACE-2 receptors, accompanied by the proteolytic cleavage of the S protein, mediated by transmembrane serine protease 2 (TMPRSS2), facilitates cell entry (Mohammadi et al., 2020). NRP1 and NRP2 act as additional viral entry factors (Daly et al., 2020). After replication, the cell disintegrates, and the virus is able to reach other cells. Then, antigen-presenting cells (APCs) recognize these viral particles and present to cytotoxic T and natural killer (NK) cells *via* the major histocompatibility complex (MHC), thus causing the production of pro-inflammatory cytokines and chemokines (Sarzi-Puttini et al., 2020). The neurological commitment of SARS-CoV-2 is associated with the expression of ACE-2 receptor in the nervous system (Iadecola et al., 2020).

One of the main causes of death from COVID-19 is acute respiratory distress syndrome (ARDS), which is characterized by a pro-inflammatory cytokine storm. Increased levels of cytokines and chemokines have been detected in the blood of patients with COVID-19; such factors include interleukin 1 beta (IL-1β), IL-6, IL-17, IL-8, C-C motif chemokine ligand 2 (CCL2)/monocyte chemoattractant protein-1 (MCP1), CCL3/MIP1α, CCL4/macrophage inflammatory protein-1 beta (MIP1β), granulocyte-macrophage colony-stimulating factor (GM-CSF), platelet-derived growth factor beta (PDGFβ), TNF, and vascular endothelial growth factor (VEGF) (Nile et al., 2020). According to Soy et al. (2020), patients with advanced age comorbidities are more likely to progress to the severe form of the disease and this risk group has a tendency towards monocytosis instead of lymphocytosis (reduction in NK cells and cytotoxic T cells), high levels of serum ferritin and D-dimer, liver dysfunction, thrombotic tendency, and disseminated intracellular coagulation (DIC), which implies the occurrence of macrophage activation syndrome (MAS). The main problems in SARS-CoV-2 infection are as follows: impaired viral shedding, low production of type I interferons (IFNs), increased neutrophils and neutrophil extracellular traps (NETs) that can contribute to viral pathogenesis, and pyroptosis, which helps in the rapid disruption of the plasma membrane and release of intracellular contents (Soy et al., 2020).

SARS-CoV-2 can also bind to TLRs, triggering their activation (Conti et al., 2020). The activation of these receptors may be followed by inflammasome activation. The activation of the inflammasome pathway may be involved in both CNS and PNS injury through the secretion of IL-1β and IL-18 (Cui et al., 2020). IL-6 is another important pro-inflammatory mediator that can be associated with damage in CNS by modulating the immune response (Mamik and Power, 2017). Furthermore, dysregulation of type I IFNs can affect both innate and acquired immunity, resulting in inflammation and immune system suppression (Conti et al., 2020). Higher levels of inflammasome-derived products and IL-6 found in the sera of the severe COVID-19 patients indicate that these factors might be a marker of COVID-19 severity (Rodrigues et al., 2020).

## COVID-19 and Neurological Commitment

Previous studies found that peripheral neuropathy may develop in patients with severe COVID-19 (Andalib et al., 2021). A systematic review of 143 original publications found that a total of 10,723 patients with a confirmed diagnosis of COVID-19 displayed features that were compatible with neurological involvement. Among them, they found 43 patients with clinical conditions affecting the PNS, mainly Guillain-Barré syndrome (Guerrero et al., 2020). Guillain-Barré syndrome is a PNS-related autoimmune condition. It has been associated with COVID-19, but the immunopathogenic mechanisms are not fully understood (Patnaik, 2021).

Pain is an important neurological symptom observed in COVID-19 patients, both in the acute phase and at later stages of the disease (McFarland et al., 2021). Studies have demonstrated that the type I IFNs involved in antiviral responses may promote virus-induced pain through actions on sensory neurons, which suggest that type I IFNs are involved in the immunopathogenesis of pain during COVID-19 (Barragán-Iglesias et al., 2020; McFarland et al., 2021), Although type I IFN is associated with an anti-SARS-CoV-2 response, several recent studies have suggested that the virus evades the type I IFN induction, which contributes to viral replication and hyperinflammatory response, which are characteristic of severe COVID-19 disease (Arunachalam et al., 2020; Zhang et al., 2020).

RNA-sequencing (RNA-seq) datasets for secretory ligands with known human dorsal root glia (hDRG) receptors demonstrated transcriptional changes modulated by SARS-CoV-2, which are able to influence nociceptor sensitization (McFarland et al., 2021). Some mediators that are modulated in bronchoalveolar lavage fluid (BALF) from patients with severe COVID-19 include the chemokines CCL2/3/4/7/8 and C-X-C motif chemokine ligand (CXCL) 1/2/6, as well as the peptide hormone epiregulin (*EREG*) and members of the ephrin A family (*EFNA1* and *EFNA5*). Analysis of single-cell RNAseq (scRNA-seq) datasets also showed a significantly upregulated expression of CCL2/3/4 and IL-1β, as well as several members of the tumor necrosis factor superfamily (TNF, TNFSF10, TNFSF12, and TNFSF13B), by macrophages from patients with severe COVID-19 infection compared to those from patients with moderate infection and/or healthy controls. Similar to the BALF findings, the authors found that peripheral blood mononuclear cells (PBMCs) from COVID-19 patients exhibit a transcriptional upregulation of prototypical signaling ligands with known hDRG receptors, including IL-1β and TNF (Singh et al., 2021). Therefore, the influence of the cytokine storm must also be considered a key factor in the development of neuropathies after severe infection and could contribute to the evolution of chronic pain after acute COVID-19 infection.

## Kynurenine Pathway and COVID-19

The elucidation of the metabolic host response is important, as some metabolites from catabolism are essential for viral infection because they contribute the nucleic acids, proteins (including capsid proteins) and membrane that are necessary for virus replication (Yan et al., 2019). Studies suggest that gut microbiota dysbiosis is involved in COVID-19 severity in patients with extra pulmonary conditions once SARS-CoV-2 infection disturbs the gut microbiota and leads to immune dysfunction with generalized inflammation disturbing the gut–brain axis (Aktas and Aslim, 2021). For this reason, the breakdown of the homeostasis of the gastrointestinal and the nervous system in response to the virus could lead metabolites such as TRYP to decrease their availability to the kynurenine pathway.

The TRYP-kynurenine pathway is altered in COVID-19 patients, as described in studies that demonstrated increased serum and or plasma levels for kynurenine and its metabolites, quinolinic acid and kynurenic acid, in COVID-19 patients (Thomas et al., 2020a; Fraser et al., 2020; Shen et al., 2020; Lawler et al., 2021). A previous study has demonstrated that an elevated kynurenic acid: kynurenine ratio was associated with increased disease severity in male patients (Cai et al., 2021).

Despite the lack of studies showing the role of the kynurenine metabolites in COVID-19 pain, many studies have shown its relationship with neuropathic pain and other painful conditions (Rojewska et al., 2016; Rojewska et al., 2018; Staats Pires et al., 2020; Jovanovic et al., 2020; Ciapała et al., 2021; Tanaka et al., 2021). Some metabolites can act as immunomodulators depending on the dose or the situation, perpetuating low-grade inflammation (Tanaka et al., 2020). However, studies show that the disturbance of the kynurenine pathway could increase the oxidative compounds or neurotoxic ligands to receptors of the excitatory glutamatergic nervous system, which damage the PNS or CNS through the broken blood–nerve or blood–brain barrier, respectively (Dantzer et al., 2008).

**Figure 1:**
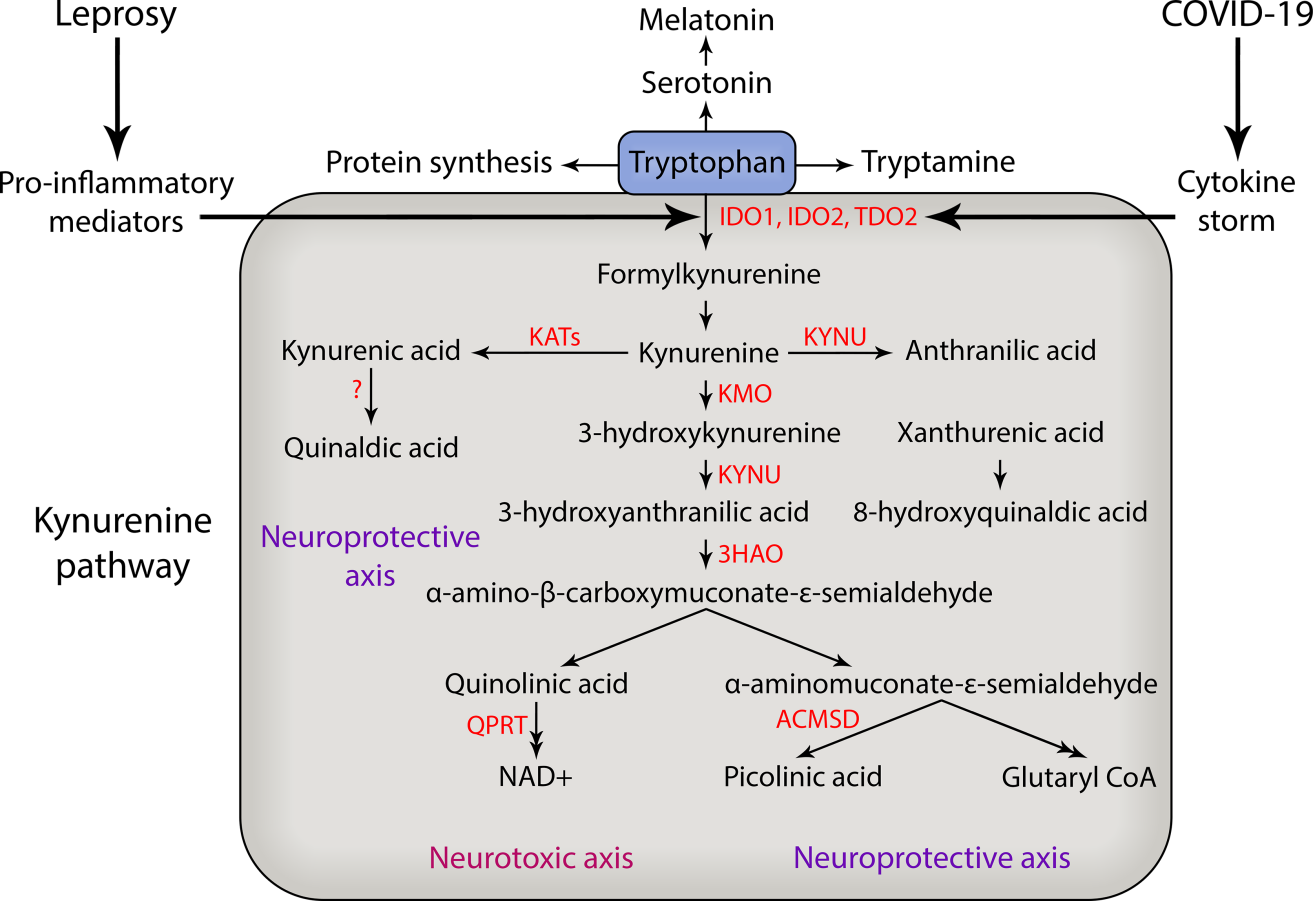
Kynurenine pathway of tryptophan degradation in leprosy and COVID-19. During the course of severe COVID-19 and leprosy immunological reactions there is an increase in production of pro-inflammatory mediators such as TNF, IL-6 and IL-1β that may activate the enzymes that catabolize tryptophan and generate neuroactive kynurenine metabolites that can contribute to peripheral nerve damage and the development of chronic pain. In addition, the cytokine storm that occurs during COVID-19 can also trigger neuropathy and reactional episodes in leprosy patients. The main kynurenine pathway enzymes are shown in red. IDO, indoleamine 2,3-dioxygenase; TDO, tryptophan 2,3-dioxygenase; KATs, kynurenine aminotransferases; KYNU, kynureninase; KMO, kynurenine 3-monooxigenase; 3HAO, 3-hydroxyanthranilic acid dioxygenase; QPRT, quinolinic acid phosphoribosyltransferase; ACMSD, aminocarboxymuconate-semialdehyde decarboxylase; NAD, nicotinamide adenine dinucleotide; CoA, coenzyme A.

Kynurenines are collectively known by the different metabolites that are produced during tryptophan catabolism, a metabolic process that is mainly governed by tryptophan rate-limiting enzymes such as IDO-1, indoleamine 2,3-dioxygenase-2 (IDO-2), and tryptophan 2,3-dioxygenase (TDO) (Campbell et al., 2014) (**Figure 1**). Tryptophan is an essential amino acid that is the precursor of many physiologically important metabolites produced during the curse of its degradation along four pathways. The kynurenine pathway is responsible for approximately 95% of overall TRYP degradation. Other pathways are hydroxylation, decarboxylation and transamination (Bender, 1983). Tryptophan is converted to *N*′-formylkynurenine by the action of either TDO mainly in liver or IDO extrahepatically (Bender, 1983; Badawy, 2017). Most previous studies on KP regulation have focused on the first and most rate-limiting enzyme, hepatic TDO. Under normal conditions the control of plasma tryptophan availability is exerted mainly, if not exclusively, by hepatic TDO. However, under conditions of immune activation, IDO assumes the major role, although TDO may also play a part. The induction of IDO by IFN-γ and other inflammatory mediators leads to depletion of TRYP and increased Kynurenine formation in cultures of monocytes and serum (Werner et al., 1988; Fuchs et al., 1990). There are two kinds of IDO, IDO1 and IDO2, both convert TRYP to kynurenine with different activity rates. IDO2 is more narrowly expressed than IDO1 and has only 3-5% enzymatic activity of IDO1 (Ball et al., 2007; Metz et al., 2007; Prendergast et al., 2014). The role of IDO2 in normal immune function is not known and due to their homology, IDO1 and IDO2 had been thought to play redundant roles in immune responses; however, recent results suggest that IDO2 may play a role in immune function distinct from IDO1 (Merlo et al., 2020).

IDO1 was described as a bactericidal effector mechanism and linked to T cell immunosuppression and tolerance. However, evidence has accumulated that suggests IDO1 also plays an important role in infections, including HIV, influenza, hepatitis B and C, and sepsis (Boasso et al., 2006; Boasso et al., 2007; Larrea et al., 2007; Ito et al., 2010; Schmidt and Schultze, 2014). In peripheral tissues, IDO1 expression takes place in dendritic cells (DCs) and macrophages, as well as microglia in the CNS, and its expression is also induced by pro-inflammatory cytokines, such as IL-6, IL-1β, IFN-γ, and TNF and by underlying infections (Campbell et al., 2014). Kynurenine (KYN) is broken down into kynurenic acid and 3-hydroxykyurenine by kynurenine aminotransferases and kynurenine3-monooxygenase (**Figure 1**). KYN is the source of different downstream metabolites of the kynurenine pathway, leading to the formation of neuroactive metabolites including kynurenic acid (KYNA), 3-hydroxykynurenine (3-HK), anthranilic acid (ANA), picolinic acid (PA), and quinolinic acid (QUIN) for example (**Figure 1**). It has been demonstrated that significant behavioral effects are induced by the intracerebro-ventricular administration of these metabolites in mice (Vamos et al., 2009).

The decline in tryptophan levels modulates the serotonin and melatonin pathway, which leads to the development of neurological disorders. Metabolites of the kynurenine pathway show diverse properties that can cause contrasting effects in the nerve system and has become an important research area in neurodegenerative disorders such as Alzheimer’s disease (AD), Huntington’s disease (HD), multiple sclerosis (MS), amyotrophic lateral sclerosis (ALS) and Parkinson’s disease (PD), due to the relationship between abnormal KP metabolite levels and these neurological diseases (Füvesi et al., 2012; Maddison and Giorgini, 2015). The comorbidities that have been associated with severe COVID-19 are aging, diabetes, hypertension, chronic lung disease, cancer, and HIV, clinical conditions, whereby the TRYP-Kyn pathway is activated.

Serum metabolic analyses of patients with COVID-19 have identified an altered tryptophan metabolism, and this change correlates with IL-6 levels. The association between IL-6 in samples from patients and COVID-19 led to an increase in KYNA, KYN, and PA (Thomas et al., 2020b). In addition, tryptophan depletion and the generation of kynurenine generate downstream signals through GCN2, WARS, mTOR and the aryl hydrocarbon receptor (AhR). Tryptophan metabolites are endogenous AhR ligands. AhR is a ligand-activated transcription factor that integrates environmental, dietary, microbial, and metabolic cues to control complex transcriptional programs in a ligand-specific, cell-type-specific, and context-specific manner. AhR plays an important role in several biological processes, including immune responses and developmental and pathological regulation (Jaronen and Quintana, 2014; Zhu et al., 2019).

AhR is involved in coordinating the entry and pathophysiology of SARS-CoV-2 (Anderson et al., 2020). SARS-CoV-2 infection activates AhR independently of IDO1, upregulating the expression of pro-viral 2,3,7,8-tetrachlorodibenzo-*p*-dioxin (TCDD)-inducible poly-adenosine diphosphate (ADP)-ribose polymerase (TIPARP) and modulating cytokines and factors such as TNF, which, in turn, activate IDO1, while AhR also increases due to the positive feedback of IDO1-AhR-IDO1 (Turski et al., 2020).

The activation of AhR induces the differentiation of IL-17-producing CD4^+^ T cells. It has already been observed that in severe SARS-CoV-2 infection there is an increase in Th17 cells and a decrease in Tregs, *via* an activation mechanism involving STAT3, JAK1, JAK2, and JAK3, and this is consequently followed by an excessive release of cytokines that can lead to severe multiple organ failure during the course of the disease (Engin et al., 2021).

Men and women show clear differences in the immune response to COVID-19. At all ages, female patients have more robust T cell activation than male patients. Men of an advanced age show a loss of T cell activation, leading to worse outcomes in the clinical course of the disease (Turski et al., 2020). Gender-specific differences were also observed in the correlations between metabolites and immune response in patients with COVID-19, in which KYNA (an AhR ligand) showed a more prominent connection to immune response in men than in women (Cai et al., 2021).

A study involving 221 biomarkers showed that ceramide metabolism, TRYP degradation, and reactions involving the consumption of NAD+, steroids, and lipids are related to the severity of COVID-19 (Marín-Corral et al., 2021). The increase in KP metabolites, such as 3-HK, seems to play a key role in disease severity (Marín-Corral et al., 2021). Further studies will determine the association between peripheral nerve damage and KYN metabolites in these patients.

## Kynurenine Metabolites and Neuroimmunomodulation

From a therapeutic perspective, KYNA is an important metabolite of KP, as it has neuroprotective properties, preventing neuronal loss following neuronal damage, and a high level of KYNA inhibits ionotropic glutamate receptors. KYNA is mainly present in astrocytes that exhibit neuroprotective property by blocking ionotropic N-methyl-D-aspartate (NMDA), α-amino-3-hydroxy-5-methyl-4-isoxazolepropionic acid (AMPA), and kainate glutamate receptors (Perkins and Stone, 1982; Schwarcz et al., 1983; Gigler et al., 2007; Vamos et al., 2009; Dantzer et al., 2011; Maes et al., 2011; Schwarcz et al., 2012). Additionally, in laboratory animals, the administration of even low amounts (nanomolar range) of KYNA into the brain can reduce glutamate levels by up to 30−40% (Zakrocka et al., 2019). Moreover, through agonistic effects on the aryl hydrocarbon receptor (AhR), KYNA regulates the immune response (Flis et al., 2016), but due to its limited ability to cross the blood–brain barrier (BBB), KYNA’s use as a neuroprotective agent is restricted (López et al., 2014).

3-HK is another neuroactive metabolite of the KP, whose production is catalyzed by kynureninemonooxygenase.3-HK from KYN may cause neuronal damage by generating free radicals. In the mammalian brain, 3-HK levels are in the nanomolar range that rise to micromolar levels in neuropathological conditions (Eastman and Guilarte, 1989; Eastman and Guilarte, 1990; Colín-González et al., 2013; Walker et al., 2013). 3-HK can be further metabolized to 3-hydroxyanthranilic acid (3-HAA), which is associated with neurological complications such as Parkinson’s disease (PD), Huntington’s disease (HD), and human immunodeficiency virus (HIV)-1-associated dementia (Vamos et al., 2009; Sorgdrager et al., 2019). The neurotoxic activity of 3-HK with respect to cells is higher in the striatum and cortex than in cells of the cerebral granule. In CNS inflammatory disease, an elevated 3-HK level is commonly detected (Colín-González et al., 2013). 3-HK has both antioxidant and pro-oxidant properties, whereas its neuropathic role is not clearly understood, as limited *in vivo* studies have been carried out to elaborate and understand its neuropathological mechanisms (Reyes Ocampo et al., 2014). While 3-HK-mediated neurotoxicity is due to hydrogen peroxide and superoxide anion production, it can also act as a free-radical scavenger and have properties that reduce lipid peroxidation (Leipnitz et al., 2007; Mithaiwala et al., 2021).

3-HAA is produced by the action of non-specific oxidases on anthranilic acid (AA), or from the oxidative cleavage of 3-HK by kynureninase. 3-HAA exhibits anti-inflammatory properties as well as both anti- and pro-oxidant properties. Its levels are elevated in patients with depression or HD (Darlington et al., 2010). The copper-dependent-superoxide- and hydrogen-peroxide-generating ability of 3-HK and 3-HAA enhance copper-associated toxicity (Ramírez-Ortega et al., 2017). 3-HAA has been found to promote apoptosis in monocytes stimulated by IFN-γ (Morita et al., 2001). Studies from human fetal nervous system culture revealed anti-oxidant and anti-inflammatory properties of 3-HAA, which are associated with the inhibition of chemokine and cytokine expression, as well as the increased expression of the antioxidant enzyme heme oxygenase-1 (Krause et al., 2011).

QUIN, generated by the enzymatic breakdown of 3-HAA, is one of the most important KP metabolites and is of huge scientific interest. Of all the KP metabolites, QUIN has the strongest evidence regarding its role in the pathology of neurological, neurodegenerative, and neuropsychiatric complications. An agonist action of QUIN toward NMDA receptors was reported (Stone and Perkins, 1981; Mithaiwala et al., 2021). The QUIN concentration generated by the KP is similar to KYNA in cerebrospinal fluid (50–100 nM) or in low micromolar concentrations (Schwarcz and Pellicciari, 2002; Vamos et al., 2009). Substantial neuronal loss is induced by intrastriatal QUIN administration (Vamos et al., 2009). Higher QUIN levels are commonly detected in patients suffering with neurodegenerative diseases such as Alzheimer’s disease (AD), PD, and HD, and in patients with HIV infections. QUIN injections in the striatum lead to a similar neurochemical and structural pathology as that observed in HD, which suggests QUIN’s potential use as a model for HD induction in laboratory settings (Heyes et al., 2001; Mithaiwala et al., 2021). QUIN was also found to disturb actin-cytoskeleton dynamics in astrocytes and neurons that perturb the transport of protein required for synaptic homeostasis (Pierozan et al., 2015). Additionally, QUIN also generates free radicals and increases lipid peroxidation, leading to increased oxidative stress, and is believed to show neurotoxic activity *via* at least nine different mechanisms, including disruption of the BBB, as well as the generation of reactive oxygen species, death of oligodendrocytes, destabilization of the cellular cytoskeleton, disruption of autophagy, and promotion of tau phosphorylation (Santamaría et al., 2001; Rahman et al., 2009). It has been supposed that, through the NMDA receptor, QUIN may trigger microglia pathways that lead to apoptosis or programmed neuronal cell death. QUIN also induces an inflammatory response by stimulating the production of pro-inflammatory mediators in astrocytes (Jacobs and Lovejoy, 2018; Pierozan and Pessoa-Pureur, 2018).

In inflammatory bowel disease, the kynurenine metabolites may be involved in the regulation of neuronal activity. Glutamate is a major excitatory neurotransmitter in the CNS and also has a significant role in the regulation of peripheral tissue function, probably by its neurotransmitter role in the enteric nerve system (Gill et al., 2000; Forrest et al., 2002). While QUIN activates, KYNA blocks the NMDA receptors present in the myenteric plexus. The activation of these NMDA receptor subtypes increases gut motility and secretion (Skerry and Genever, 2001; Forrest et al., 2002).

The increase in KP activity in the PNS can be detected by the elevated serum KYN/TRYP ratio, which is usually found in several psychiatric and neurological disorders (Schwarcz et al., 2012; Zunszain et al., 2012). Some metabolites, such as KYN, 3-HK, and AA, cross the BBB, while KYNA, QUIN, and 3-HAA cannot cross, or can only cross to a limited extent (Gobaille et al., 2008; Schwarcz et al., 2012). Large neutral amino acid transporter 1 (LAT-1), as well as organic anion transporters 1 and 3, play an important role in the transport of peripheral KYN through the BBB. Over 60% of KYN in the CNS are transported from the peripheral circulation (Gobaille et al., 2008; Sekine et al., 2016; Walker et al., 2019). There is little information regarding the involvement of the KP in the PNS, but recent findings in leprosy pathogenesis have contributed new evidence of the involvement of TRYP metabolites in neural damage in the PNS.

## Kynurenines in Peripheral Neuropathy During COVID-19

Several studies have identified a number of neuropathologies associated with SARS-CoV-2, including pain-related conditions (McFarland et al., 2021) but recent evidence suggests that SARS-CoV-2 does not cause viral neuropathy (Finsterer et al., 2021). At present, there is evidence that neural damage during COVID-19 is associated with secondary immune mechanisms (Finsterer et al., 2021). It is hypothesized that the systemic hyperinflammation seen in severe COVID-19 has the potential to contribute to nociceptor sensitization.

IFN-γ is, at present, seen in significantly higher concentrations in sera from SARS-CoV-2-infected patients. The activation of IDO1 by interferons may help explain the observations of higher concentrations of kynurenine and its catabolites in infected patients. Proteomic analysis revealed that both QA and 3-HK are increased in samples from infected patients (Lawler et al., 2021). Both QA and 3-HK show neurotoxic properties *in vitro*. In addition, the ratio between both metabolites (QA and 3-HK) and the neuroprotective KYNA was also found to increase in the SARS-CoV-2 infected group, both of which have specifically been referred to as neurotoxic ratios in the literature.

## Kynurenines in Leprosy Pathogenesis

A recent transcriptomic analysis demonstrated that *IDO1* expression could be used as a biomarker to discriminate skin lesions of leprosy patients from controls affected by other dermal conditions, such as granuloma annulare (Leal-Calvo et al., 2021). IDO1 expression and activity are increased in multibacillary lepromatous when compared with the paucibacillary patients (de Souza Sales et al., 2011). *In vitro* studies demonstrated that *M. leprae* infection leads to an increase in IDO1 expression and activity in human monocytes by an IL-10-dependent mechanism (Moura et al., 2012) and that IDO1 activity is associated with increased bacterial viability inside host cells (de Mattos Barbosa et al., 2017).

The increase in IDO1 enzymatic activity has been described to occur by different cytokines such as IFN-γ, IL-6, IL-10, and TGF-β (Orabona et al., 2008; Pallotta et al., 2011; Moura et al., 2012; Andrade et al., 2015; Li et al., 2016). It has recently been shown that TLR2 activation is necessary for IDO1 induction in monocyte-derived dendritic cells (mDCs). Moreover, mycobacterial fractions could differentially modulate IDO1 expression and activity. While the membrane fraction of *M. leprae* induced the production of pro-inflammatory cytokines TNF and IL-6, the soluble fraction induced an increase in IL-10 in mDCs. The co-culture of mDCs with autologous lymphocytes induced an increase in regulatory T cell (Treg) frequency in MLSA-stimulated cultures, showing that *M. leprae* constituents may play opposite roles that could possibly be related to the dubious effect of IDO1 in the different clinical forms of disease (Oliveira J. A. P. et al., 2021).

Although the evaluation of sera from multibacillary patients demonstrated greater IDO1 activity when compared with sera from paucibacillary patients, there was higher variability in the multibacillary group. Higher IFN-γ-dependent IDO1 expression and activity are observed in cells from multibacillary patients who have developed an acute inflammatory episode (type-1 or reversal reaction) (Andrade et al., 2015). Patients with a type 1 reaction show an improvement in neural damage, maybe due to the higher levels of pro-inflammatory cytokines TNF and IFN-γ. These data suggest that, in multibacillary patients, IDO1 activity and activation of the kynurenine pathway should not be restricted to the induction of tolerance in skin cells but may be involved in the pathogenesis of neural damage. Future studies should elucidate whether the increased IDO1 activity and increased levels of kynurenine metabolites in serum can be correlated with leprosy neuropathy in multibacillary patients. This could be important for the identification of a neuropathy biomarker in multibacillary patients, since neuropathy in this group frequently evolves as a silent neuropathy, and, therefore, early diagnosis is fundamental for avoiding disabilities and incapacities.

## Conclusion

**Figure 2:**
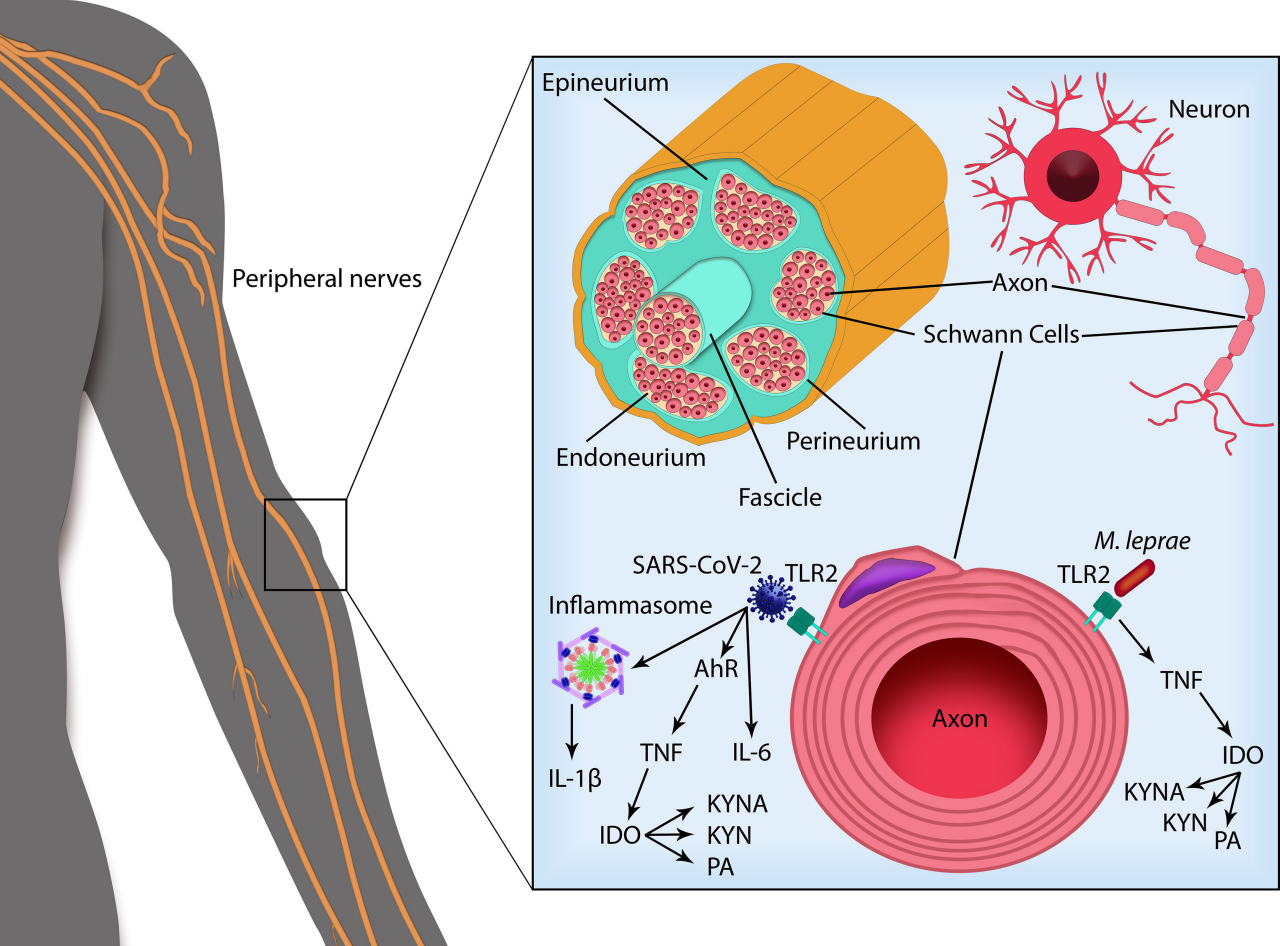
SARS-CoV-2 and *Mycobacterium leprae* infection in peripheral nerve Schwann cells. Peripheral nerves are composed offascicles delimited by the perineurium and enveloped by the epineurium. Inside the nerve fascicles, surrounded by the endoneurium, the axons from each neuron are encircled by Schwann cells (SCs) that form the myelin sheath. SARS-CoV-2 infection activates the transcription factor aryl hydrocarbon receptor (AHR), leading to TNF production. SARS-CoV-2 can also bind TLRs triggering their activation and subsequent production of pro-inflammatory cytokines as IL-6 and IL-1β, cleaved by caspase-1 following inflammasome activation. Recognition of *M. leprae via* TLRs in the SCs leads to the production of TNF. *M. leprae* infection in myelinating SCs can also induce apoptotic cell death by a mechanism dependent on TNF and TLR2, degradation of myelin sheath and peripheral nerve fibrosis. TNF (as well as IL-6 and IL-1β) activates the enzyme indoleamine 2,3-dioxygenase (IDO) that catabolizes tryptophan *via* kynurenine pathway. Formation of neuroactive metabolites of kynurenine (KYN) such as kynurenic acid (KYNA) and picolinic acid (PA) can contribute to nerve damage. SARS-CoV-2 and *M. leprae* co-infection may trigger a higher pro-inflammatory state leading to increased neuropathy and possibly triggering leprosy reactional episodes.

Although there are no specific studies evaluating the immunopathogenesis of leprosy-COVID-19 co-infection, in both diseases, the KYN metabolites are associated with a worsening in clinical conditions, which could contribute to neural damage, since some KYN metabolites have been described as neurotoxic agents (**Figure 2**). Determining the role of KYN metabolites in the pathogenesis of both diseases, individually and in co-infected patients, may contribute to the development of new diagnostic and therapeutic strategies.

The identification of effective and non-toxic IDO1 inhibitors designed to treat infectious diseases is an urgent need. In recent years, enormous attempts have been made to advance the IDO1 inhibitors, resulting in a diverse range of selective and potent IDO1 inhibitors. Research is still on-going, motivated by the fact that these inhibitors have already been used in the treatment of some types of cancers. The IDO1 inhibitors have therapeutic utility in various diseases and, in the near future, may be of use in the treatment of peripheral neuropathy observed in patients with leprosy, COVID-19, or both.

## Author Contributions

JO, MMA, AR, MdMB, and RP wrote the manuscript. RP and MdMB made the figures. MJ, MOM, and RP provided intellectual output in the manuscript. All authors contributed to the article and approved the submitted version.

## Funding

We thank the CAPES, FAPERJ (E-26/201.176/2021 (260734), Fiocruz (INOVA Geração de conhecimento - 52301630878), and CNPq (312802/2020-0) funding institutions for all their financial support.

## Conflict of Interest

The authors declare that the research was conducted in the absence of any commercial or financial relationships that could be construed as a potential conflict of interest.

## Publisher’s Note

All claims expressed in this article are solely those of the authors and do not necessarily represent those of their affiliated organizations, or those of the publisher, the editors and the reviewers. Any product that may be evaluated in this article, or claim that may be made by its manufacturer, is not guaranteed or endorsed by the publisher.
